# Cincinnati Eye Infirmary

**Published:** 1836

**Authors:** 


### V.—Cincinnati Eye Infirmary.

The readers of the Western Journal have at various times seen in it, notices of the Cincinnati Eye Infirmary. Although incorporated by the General Assembly of Ohio, no endowment of any kind was bestowed upon it by that honorable body, and the Editor of this Journal, as its Physician and Surgeon, has been obliged to perform the duty of providing, as well as he could, the necessary accommodations for those indigent persons, laboring under chronic affections of the eye, who from time to time were attracted to it. He is happy to say, that the embarrassments under which he has so long labored, in reference to these accommodations, have at length been removed, and that by an arrangement with the Medical Faculty of the Cincinnati College, the Eye Infirmary makes a department of the *Cincinnati Hospital*, lately founded by them, and that the means of boarding, lodging and nursing patients afflicted with maladies of the eye, whether rich or poor, have been provided, and that any number who may apply can be received.

